# Virtual race transformation reverses racial in-group bias

**DOI:** 10.1371/journal.pone.0174965

**Published:** 2017-04-24

**Authors:** Béatrice S. Hasler, Bernhard Spanlang, Mel Slater

**Affiliations:** 1 Sammy Ofer School of Communications, Interdisciplinary Center (IDC) Herzliya, Israel; 2 Experimental Virtual Environments (EVENT) Lab for Neuroscience and Technology, Department of Clinical Psychology and Psychobiology, University of Barcelona, Barcelona, Spain; 3 Institució Catalana de Recerca i Estudis Avançats (ICREA), Barcelona, Spain; 4 Department of Computer Science, University College London, London, United Kingdom; University of Bologna, ITALY

## Abstract

People generally show greater preference for members of their own racial group compared to racial out-group members. This type of ‘in-group bias’ is evident in mimicry behaviors. We tend to automatically mimic the behaviors of in-group members, and this behavior is associated with interpersonal sensitivity and empathy. However, mimicry is reduced when interacting with out-group members. Although race is considered an unchangeable trait, it is possible using embodiment in immersive virtual reality to engender the illusion in people of having a body of a different race. Previous research has used this technique to show that after a short period of embodiment of White people in a Black virtual body their implicit racial bias against Black people diminishes. Here we show that this technique powerfully enhances mimicry. We carried out an experiment with 32 White (Caucasian) female participants. Half were embodied in a White virtual body and the remainder in a Black virtual body. Each interacted in two different sessions with a White and a Black virtual character, in counterbalanced order. The results show that dyads with the same virtual body skin color expressed greater mimicry than those of different color. Importantly, this effect occurred depending on the virtual body’s race, not participants’ actual racial group. When embodied in a Black virtual body, White participants treat Black as their novel in-group and Whites become their novel out-group. This reversed in-group bias effect was obtained regardless of participants’ level of implicit racial bias. We discuss the theoretical and practical implications of this surprising psychological phenomenon.

## Introduction

Several studies have recently shown that multisensory stimulation based on the rubber hand illusion [1] or immersive virtual reality (VR) may be used to substitute a person’s arm by a rubber arm or their full body by a life-sized virtual body, resulting in a strong perceptual illusion that the body part or full virtual body is their own. These are examples of ‘body ownership illusions’ [2–4]. It has further been shown that attributes of the surrogate body can have an impact on participants’ perception and implicit attitudes. For example, embodying adults in a child virtual body results in overestimation of object sizes and implicit self-identification as being more child-like [5], compared with being embodied in an adult-shaped body of the same size as the child. Of particular interest is that when the participants are White and the illusorily owned rubber arm or virtual body is black then this reduces their implicit racial bias towards Black people [6–8]. Previous results in this area have relied on Implicit Association Tests (IAT) [9], where, for example, bias is shown by participants being more rapidly able to pair negative attributes with Black faces and positive attributes with White faces than the other possible combination. After the embodied experience (with perceptual ownership of a black rubber arm or full virtual body) this bias decreases on the average [6, 7, 10, 11].

In this paper we show that there is a strong behavioral impact of such virtual embodiment that occurs in a dyadic interaction between White participants embodied in a White or Black body, and a Black or White virtual human. In real-world interactions, people of the same race (or generally same social ‘in-group’) are far more likely to engage in mimicry of their partner than when they are of a different race (or generally when one is perceived as ‘out-group’ by the other) [12]. We show that in VR the skin color of participants’ *virtual body* rather than their real body influences who they mimic more. Since mimicry is a non-conscious behavior that signifies social rapport this shows how actual behavior may be impacted through such virtual embodiment, beyond what can be found from implicit associations. In the next sections we provide the background on racial (in-group and out-group) categorization and on mimicry, before describing the experimental study.

People identify themselves not only as individuals but also as members of social groups to which they belong (i.e., a set of ‘in-groups’). In-group members are perceived as similar to the self and are differentiated from dissimilar others (i.e., ‘out-groups’) [13]. Such classifications into ‘us’ versus ‘them’ occur spontaneously during social interactions, and influence interpersonal perceptions and behaviors. People generally show greater attachment to and preference for people in their in-group than in their out-group, which is referred to as in-group bias or in-group favoritism [14]. While any social category that is salient during an interaction may lead to in/out-group distinction, race represents a particularly potent cue to group membership due to its visual salience [15]. Race categorization occurs within milliseconds; even faster than the detection of other physical characteristics, such as age and gender [16, 17].

Interracial interactions are especially prone to in-group bias. It has been repeatedly shown that own-race faces are more accurately encoded and better remembered than other-race faces, see the review by Meissner and Brigham [18]. Besides these perceptual differences, racial in-group members tend to be evaluated more favorably than racial out-group members. Such own-race preferences have been demonstrated across the lifespan. Infants as early as three months old have been found to prefer own-race faces over faces of other racial groups [19]. These biases may correspond to differing brain activity when observing racial in-group and out-group members [20, 21].

Observing the actions of others typically activates brain areas similar to the areas that are activated when performing these actions oneself [22]. Some actions are not only mentally simulated but also actually mimicked. The mere perception of these actions increases the likelihood of behaving in a similar way [12]. This includes the spontaneous imitation of postures, gestures, facial expressions, and mannerisms, which usually occurs without awareness. Perception-action coupling has been claimed to be an evolutionary beneficial mechanism that increases liking and rapport between interaction partners [23]. However, this appears to be reserved for interactions between in-group members. For example, Gutsell and Inzlicht [24] showed in an EEG study that only the perception of actions performed by own-race models activated the motor system. When perceiving actions of other-race models the motor system was less active. These findings are consistent with behavioral studies that have shown that individuals tend to mimic their in-group members to a greater extent than their out-group members [25–27]. Rates of mimicry have been found to be particularly low for highly prejudiced individuals [27] and in interactions with disliked others [28] and stigmatized people [29]. Reduced motor resonance with out-group members has important consequences for interracial interactions. It is associated with lower levels of interpersonal sensitivity and empathy [30, 31], which may hinder the establishment of positive interracial relationships.

Although in-group bias occurs automatically as a consequence of social categorization, research has shown that both the self-concept [32] and the evaluative processes in intergroup perception are dynamic and malleable [33]. A common method to alter in-group bias is perspective-taking, which typically requires individuals to imagine how it would be to be a member of the out-group. Taking the other’s perspective has been claimed to increase self-other overlap and foster the transfer of positive self-evaluations to the out-group, thereby reducing in-group bias [34]. Despite its popularity and demonstrated success in changing attitudes and discriminatory behavior [35, 36], perspective-taking has its limitations. It requires a capability for mental imagery, cognitive effort and motivation from the individual in order to be effective. Moreover, when applied during an interracial interaction, perspective-taking may have only small effects and might even have negative consequences for highly prejudiced individuals [37].

In the experiment described in this paper we made perspective-taking explicit through using immersive VR. Instead of *imagining* how it would be to be someone else, we actually put people into a situation of ‘being’ a member of the out-group through virtual embodiment. Embodiment in a life-sized virtual body that is perceived from first person perspective as visually substituting the person’s real body has been shown to lead to an illusion of ownership over the virtual body [38]. Moreover, through the application of real-time motion capture, the virtual body can be programmed to move synchronously and in correspondence with real body movements. In this case there is also strong agency with respect to the virtual body, so that participants attribute actions of the virtual body to themselves [39]. These effects do not occur when the virtual body movements are asynchronous with respect to real movements—e.g. [5, 39, 40].

Virtual embodiment was first used in the context of racial bias in Groom, Bailenson, et al. [41]. This placed participants in a Black virtual body for 60–75 s, that had head movements synchronous with real movements that participants could see in a virtual mirror. The setting was a social situation involving the participant being in a job interview. An IAT was used to assess racial bias, and the results showed an increase in racial bias against Black people as a result of the manipulation. However, subsequent studies found the opposite effect. Using the rubber hand illusion with a black rubber arm Farmer, Tajadura-Jimenez, et al. [11] found some evidence supporting the possibility that subjective ownership over a Black rubber hand in the rubber hand illusion may reduce implicit bias, a result later taken up by Maister, Sebanz, et al. [7] who found a reduction in implicit bias due to such ownership. Similarly Peck, Seinfeld, et al. [6] using full body virtual embodiment in a virtual body also found a reduction in implicit bias. Banakou, PD, et al. [10] found the same effect even though the implicit bias was measured one week after the VR exposure. Some of these results have been reviewed by Maister, Slater, et al. [8], and we return to this topic in the Discussion.

While earlier studies have been concerned with attitudinal change concomitant with the type of virtual body, the focus of the current research lies in the *behavioral* consequences of VR-based manipulations of racial embodiment. First, we examine whether there are differences in experienced body ownership with respect to embodiment of White people in a White or Black virtual body. Second, we examine the influence of this embodiment on changes in racial bias using the IAT. Third, and most importantly we test how racial embodiment influences social mimicry in a simulated interracial interaction.

**Fig 1:**
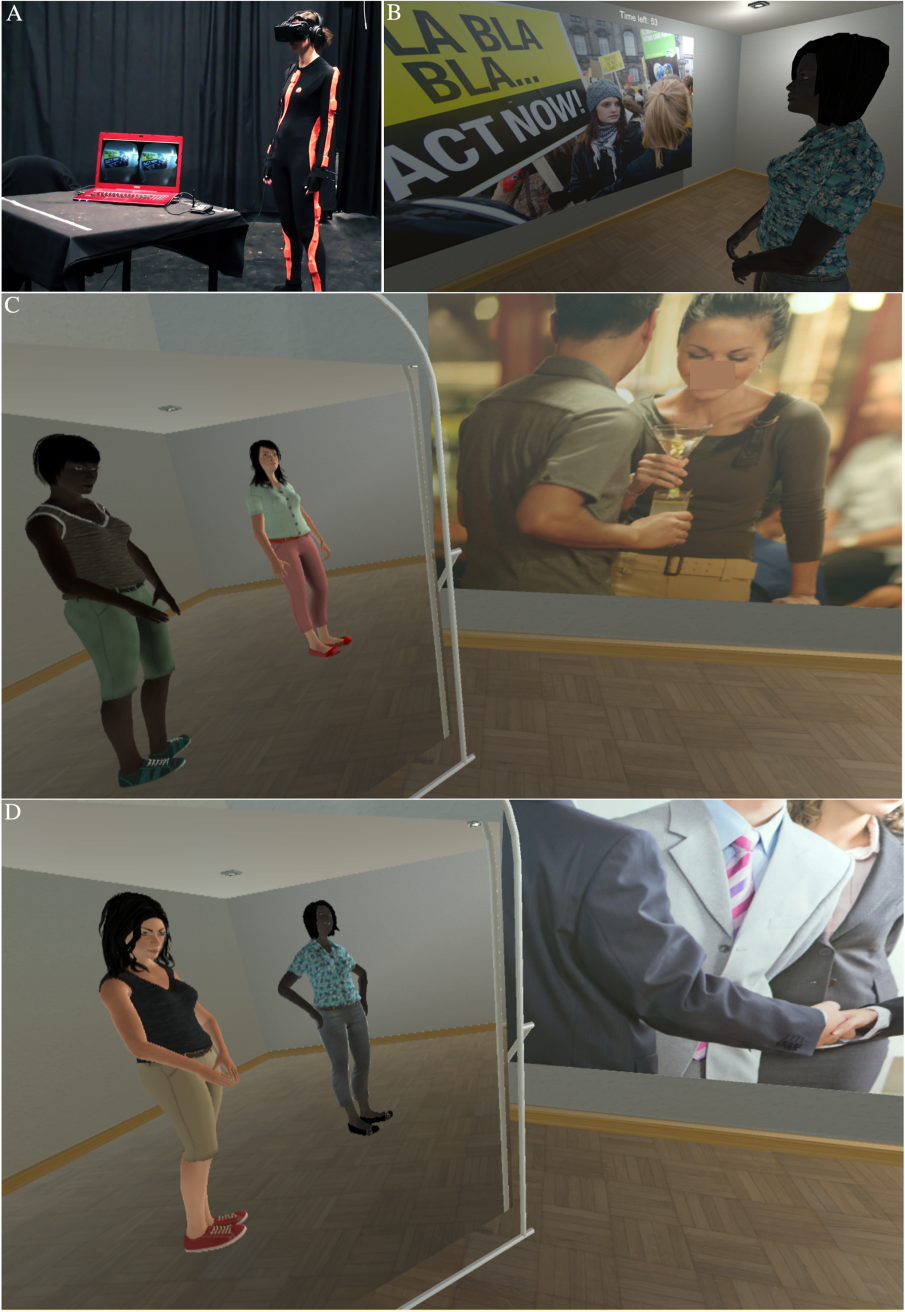
The scenario (A) The physical setup showing the participant wearing a full body motion capture suit and the head-mounted display (B) The participant sees a picture displayed on the front wall and her virtual (Black) partner to her right. (C) The participant is embodied as Black, her virtual (White) partner is to her right, and she can see herself and her partner in the mirror (D) The participant is embodied as White and can see herself and her (Black) partner in the mirror. The virtual interaction partners are the same apart from skin color and clothing, as described in Methods.

The experimental study was conducted with 32 White female participants. Each participant was embodied with a virtual body seen from first person perspective with full visuomotor synchrony. They interacted with another virtual human character for 6 minutes. The form of interaction was that in turn each (the real and virtual person) had to describe a series of pictures that they saw on the virtual wall–the Picture Description Task. There were two binary factors: Own Body (Self Black or Self White) and Other Body (Other Black or Other White). Half of the participants were randomly assigned to the Self Black and half to the Self White condition. Each participant repeated the study twice, always with the same Own Body skin color. However, the skin color of the Other Body was different in the two trials, the order counterbalanced across all participants. Hence Own Body Color was a between groups factor and Other Body Color was within groups. Details are given in Methods. The experimental setup can be viewed in S1 Video, and is illustrated in Fig 1.

A questionnaire used in previous experiments—e.g. [39]—was given immediately after the exposure to elicit the degree of body ownership and agency (Table 1). The first two questions are a direct assessment of the illusion of body ownership, and NotMe is to check for consistency with the first two, where for a strong illusion of body ownership we would expect high scores on the first two and low scores on NotMe. MBodies can be considered as a control question for body ownership, since high body ownership should not entail the feeling of having two bodies—only the virtual body should be salient. The last two questions are assessments of agency. Additional questions on the participants’ explicit attitudes towards the virtual Other are discussed in S1 Text. A racial Implicit Association Test was given prior to (PreIAT) and after (PostIAT) the VR experience. The PreIAT was administered 2–3 days before the first VR exposure and the PostIAT after the second exposure. (See Methods for details of these measures). The degree of mimicry of the Other (virtual) person was measured as a count of the number of mimicked behaviors coded by two independent coders. We refer to this as nmimicry.

**Table 1:** Body ownership and agency questionnaire each score is on a 1–5 Scale, where 1 = completely disagree, 5 = completely agree.

Variable Name	Statement
MeMirror	Although the virtual body did not look like me, I had the sensation that the virtual body I saw in the mirror was mine.
MeDown	Although the virtual body did not look like me, when I looked at my body I had the sensation that it was mine.
NotMe	I felt that the virtual body was not mine.
MBodies	During the experiment there were moments when I felt I had more than one body.
Agency	I felt that I controlled the avatar as if it were my body.
MyMovements	The virtual body moved according to my movements.

## Results

### Body ownership and agency

**Fig 2:**
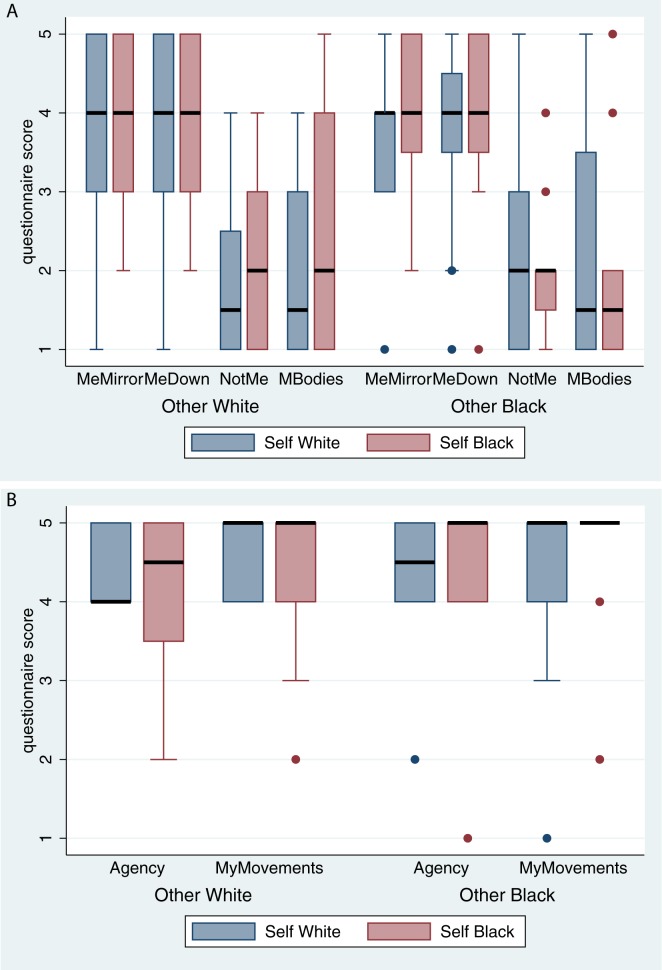
Questionnaire responses on body ownership and agency. (A) The questions related to ownership. (B) The questions related to agency. The medians are shown as the thick black horizontal lines, and the interquartile ranges (IQR) are the boxes. The whiskers extend from the quartiles ±1.5×IQR within the range of the data. Values outside of these ranges are shown as individual outliers.

The box plots of the questionnaire scores from Table 1 are shown in Fig 2. The results regarding body ownership (Fig 2A) suggest that the conditions (OwnBody, OtherBody) do not influence any of the scores. Additionally the two questions that directly assess body ownership positively (MeMirror and MeDown) have high scores under all conditions—the median being 4 out of a maximum of 5, in every case—whereas the other questions (NotMe and MBodies) have low scores—their medians are at most 2.

In order to test the first point one overall score for the ownership illusion was constructed using polychoric principle components analysis [42]. This method specifically accounts for the fact that the observed variables are ordinal, and constructs underlying continuous latent variables corresponding to the principle components. The first principle component accounts for 69% of the variation in the scores and the first two principle components together account for 90%. We call the corresponding latent variables Ownership1 and Ownership2 respectively. Ownership1 is highly correlated with each of the questionnaire scores and Ownership2 only with MBodies (see Table 2). A mixed effects regression of Ownership1 on Own Body + Other Body + Own Body × Other Body with robust standard errors reveals no significant terms. The interaction term has z = 1.36, P = 0.173, and eliminating the interaction term the main effects have Own Body (z = 0.25, P = 0.802) and Other Body (z = -0.65, P = 0.516). Similarly there are no effects for Ownership2.

**Table 2:** Body ownership questionnaire scores. Spearman correlation coefficients (ρ) and corresponding significance levels (P) between the original scores and the principle component latent variables. A significance level 0.0000 indicates P < 0.00005.

	MeMirror	MeDown	NotMe	MBodies
Ownership1	ρ = 0.81 (0.0000)	ρ = 0.84 (0.0000)	ρ = -0.88 (0.0000)	ρ = -0.56 (0.0000)
Ownership2	ρ = 0.21 (0.102)	ρ = 0.16 (0.193)	ρ = 0.15 (0.252)	ρ = 0.85 (0.0000)

Since the factors (Own Body, Other Body) do not influence the questionnaire scores, Wilcoxon matched-pairs sign-rank tests are used to compare the overall scores irrespective of these factors. The results in Table 3 show that there is no difference between MeMirror and MeDown, and that each of these is quite different from the consistency question (NotMe) and the control question (MBodies), supporting what can be seen in Fig 2A.

**Table 3:** Differences between the body ownership questionnaire scores. Wilcoxon matched-pairs sign-rank tests z with corresponding significance level (P). A significance level 0.0000 indicates P < 0.00005.

	MeMirror	MeDown	NotMe	MBodies
MeMirror		z = 0.281 (0.778)	z = 5.505 (0.0000)	z = 5.780 (0.0000)
MeDown			z = 5.134 (0.0000)	z = 5.565 (0.0000)
NotMe				z = -0.404 (0.686)

The MyMovements and Agency questions (Fig 2B) have very high scores, the median being 5, and the 25^th^ percentile at least 4 in all conditions. These are in fact only a test of the workings of the system, since through the real-time motion capture it was the case that the movements of the virtual body corresponded well to the real movements of the participants.

### Implicit association tests for racial bias

A racial IAT was administered before the first (PreIAT) and after the second (PostIAT) VR exposure. Since each participant experienced both Other Black and Other White conditions, only the between groups factor Own Body is relevant (Self Black and Self White, n = 16 in each group). The means ± standard errors of PreIAT are 0.60 ± 0.08 for Self White (t(15) = 7.69, P < 0.00005 for the two-tailed test that the mean is 0), and 0.56 ± 0.08 for Self Black (t(15) = 6.98, P < 0.00005). The hypothesis that the two means are equal is not rejected (t(30) = 0.32, P = 0.75). The change in IAT (dIAT = PostIAT—PreIAT) after the two VR exposures are similarly not different: -0.09 ± 0.09 for Self White and -0.11 ± 0.17 for Self Black (t(30) = 0.89, P = 0.449).

**Fig 3:**
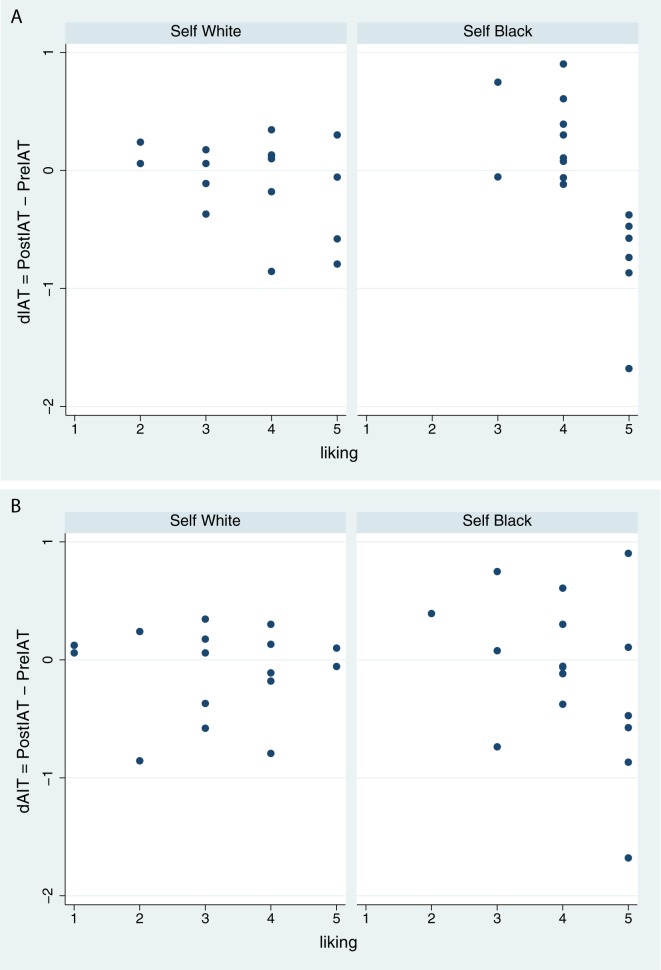
Scatter diagram of change in IAT on liking by own body. (A) After interaction with the Black virtual partner. (B) After interaction with the White virtual partner.

After each trial participants were asked ‘How much did you like the other person (with whom you had carried out the task)?’ on a 1 (not at all) to 5 (a lot) scale (see also S1 Text). We refer to this as variable ‘Liking’. Fig 3A shows the scatter plot of dIAT on Liking by the factor Own Body amongst those who had interacted with the Black virtual partner in the trial that they had just completed. Those embodied in the Black body show that decrease in dIAT is associated with greater Liking, whereas this is not the case for those embodied in the White body. ANCOVA of dIAT on Own Body + Liking + Own Body × Liking shows that the negative slope for those in the Black Body is significantly different from the White Body case (F(1,28) = 9.11, P = 0.005, Partial η^2^ = 0.25), with overall R^2^ = 0.43). The residual errors of the fit are compatible with normality (Shapiro-Wilk test P = 0.23). To check that this is not caused by the lowest extreme point in the Self Black condition, when we eliminate this point F(1,27) = 6.67, P = 0.016, Partial η^2^ = 0.20, with overall R^2^ = 0.39 (Shapiro-Wilk P = 0.88). By contrast Fig 3B shows that in the situation of interaction with the White virtual partner there is no association between dIAT and Liking: the equivalent statistics are F(1,28) = 1.93, P = 0.18, Partial η^2^ = 0.06, with overall R^2^ = 0.10 (Shapiro-Wilk P = 0.42). Of course if the outlying point is eliminated then the fit is even worse (e.g., R^2^ = 0.05). Further consideration of the impact of the variable ‘Liking’ is given in S1 Text.

### Mimicry

**Fig 4:**
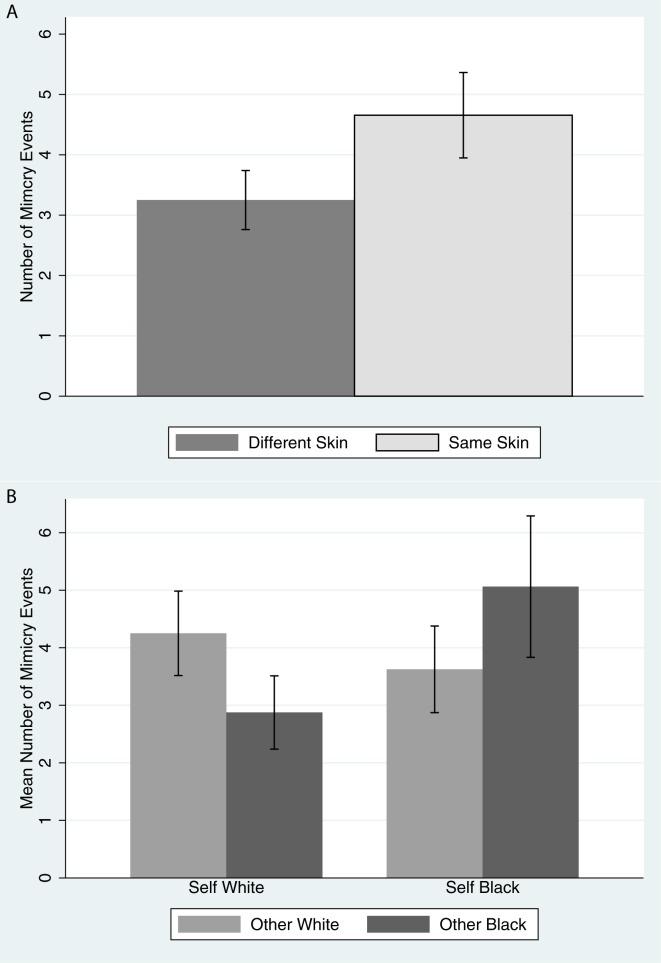
Bar charts of means and standard errors of the number of mimicry events by conditions (A) Comparing when Self and Other bodies have the same or different skin color (B) by the Self and Other Body conditions.

The mimicry response variable is our major variable of interest. Operationally it is a count of the number of mimicry events that occurred in the different conditions (see Methods). Fig 4A shows the mean and standard errors of the mimicry counts (nmimicry) by the match of skin color between Own Body and Other Body (Same Skin, Different Skin). It can be seen that the mean nmimicry is greater when the skin color of the Own Body and the Other Body are the same. However, the bar charts do not take into account the repeated measures over the participants. Since we are working with a count variable the appropriate error distribution is Poisson. A mixed effects regression, using the Stata 14 function ‘mepoisson’ with robust standard errors, shows z = 3.22, P = 0.001 for the test of no difference between Same Skin and Different Skin. The 95% confidence interval for the difference between the means of Same Skin and Different Skin is 0.14 to 0.58.

Fig 4B shows the result in more detail with respect to the skin colors. It is again clear that the amount of mimicry was greatest when the two bodies had the same skin color compared to when they were different. A mixed effects (Poisson) regression of nmimicry on Own Body + Other Body + Own Body × Other Body shows a significant interaction effect (z = 3.14, P = 0.002) indicating that at least one combination of Own Body and Other Body is significantly different from the others. Our specific interest is to compare the amount of mimicry when Own Body is White and the Other Body is White compared to Black, and when Own Body is Black and the Other Body is Black compared to White. In order to test this we used multiple simultaneous contrasts (Sidak method) with the results shown in Table 4. The results support the idea that the amount of mimicry is greater when the participant is embodied as Black and the virtual partner is Black than when the virtual partner is White, and similarly when Own Body is White greater mimicry when the Other Body is White compared to Black (though this is supported at a lesser level of significance).

**Table 4:** Contrasts for (own body, other body), sidak method of multiple comparisons S = Self, O = Other, W = White, B = Black. Results are on a log scale due to poisson regression.

Terms	Contrast	S.E.	P	95% CI
(SW,OW)–(SW, OB)	0.39	.19	0.069	-0.02 to 0.81
(SB, OB)–(SB, OW)	0.33	.14	0.030	0.03 to 0.64

Finally it should be noted that the IAT scores show no significant influence if added to the models discussed in this section. Hence the mimicry results occur irrespective of the Pre- or PostIAT scores.

## Discussion

The experiment has demonstrated three results that we discuss in turn. First, the participants had strong body ownership illusion over the virtual body, irrespective of skin color (Fig 2). This is in line with previous results on full-body ownership in immersive VR. For example, in [6] it was found that there was a strong body ownership illusion (all participants White) over a White, Black and purple body. In [5] there was strong body ownership of adults over a (five year old) child body, provided that there was visuomotor synchrony and correspondence between real and virtual body movements. Experiencing the body from first person perspective [38, 43] is one critical factor for body ownership, and when movement of the real body is allowed then visuomotor synchrony with corresponding movements is also necessary [39, 40]. However, this result stands in contrast with [11] who found that White participants reported a stronger sense of ownership over a white than a black rubber hand. It is possible that holistic racial transformations using full-body ownership illusions are easier to achieve than the illusion of ownership of a single artificial body part. This result on full body ownership has now been replicated four times: the study by Peck et al. [6], and two experiments reported in [10], together with the results in this paper.

Second, we found that there was no change in implicit racial bias as measured by the IAT simply as a result of the embodiment, i.e., the mean changes are the same whether the participants were embodied in the White or Black body. However, this study is different from those described in [6, 10] since there was a social interaction that participants could have experienced as negative or positive depending on their own individual preferences, keeping in mind that the virtual partner’s verbal and nonverbal behaviors were standardized and cross-balanced across conditions (see Methods).

This finding stands apart from all but one of the previous experiments that have used multisensory integration to achieve embodiment of White participants with a Black body (or body part in the case of the Rubber Hand Illusion) [6, 7, 10], where an overall and significant reduction in implicit bias has consistently resulted from the embodiment. The exception was Groom, Bailenson, et al. [41] which found that implicit racial bias increased. Apart from the many methodological differences between that study and the others, there is one common factor with the current study—which is that there was a social interaction involved. In the case of [41] this was in the situation of a job interview, which is associated with an exacerbation of implicit bias [44, 45]. In the current study the scenario involved a social interaction that, in itself, was neutral, but where participants may have liked their (virtual) partner to a lesser or greater degree. Intergroup contact theory argues that positive interaction with members of an ‘out-group’ tends to reduce bias [46–48]. This would imply that participants in our study who happen to have liked their virtual partner should show a greater reduction in implicit bias than those who did not. Although this was not one of our hypotheses prior to this experiment we found that for those embodied as Black the extent to which they liked their virtual partner was associated with a reduction in implicit bias. We cannot know the direction of causality but this is partially consistent with the Contact hypothesis [47, 48], except according to that racial bias should have decreased also for those who interacted with the Black virtual character while in the White body the more that they liked that character.

Turning now to the main purpose of our research, this was to address the third point, whether the degree of mimicry is greater for same-race dyads compared to different-race dyads. Interestingly, we have found that this effect occurs depending on the *virtual body’s race*, *not participants’ actual racial group membership*. When embodied in a Black virtual body, White participants treated their Black virtual partner as if she was a member of their in-group (i.e., more favorably as indicated by increased mimicry), and the White partner was treated like an out-group member (decreased mimicry). This finding demonstrates the plasticity of racial self-categorizations and the malleability of the racial in-group bias [14].

This result may be caused by perceptual similarity between the virtual bodies. People are generally more attracted to and more likely to form relationships with others who are similar to themselves in terms of psychological or physiological attributes than with dissimilar others [49]. The desire to affiliate with similar others has been found to activate automatic social behaviors, such as mimicry as an unconscious attempt to increase rapport and closeness between interaction partners [50]. Skin color can provide a strong cue for self-similarity [15]. When being immersed in VR, the skin color of one’s virtual body is visually salient while the physical body is not visible. Hence it is likely that the properties of the virtual body rather than participants’ physical body determine who is perceived as self-similar. However, it is clearly culturally salient ‘race’ rather than simply ‘being different’ that is one of the roots of implicit bias, since Avenanti, Sirigu, et al. [51] found that individuals will empathically react to the pain inflicted on others when they are in the same racial in-group or when they are quite different (e.g., having violet colored skin) but not when they are clearly representative of a racial out-group.

Reversed in-group bias may be explained by a process called egocentric social projection [52], which has been found to be activated even in conditions of minimal group membership; that is, when people are assigned to a novel in-group according to arbitrary criteria, such as shirt color [53]. People tend to use the self as a source of information to give meaning to a novel in-group (i.e., self-anchoring; [54]). As people typically evaluate themselves positively, these positive evaluations are projected onto the novel in-group. By altering people’s self-views, they also change their perceptions of the in-group [55]. Hence, when we put White participants into a Black virtual body, the novel racial category becomes associated with the self, and they automatically transfer their positive self-evaluations onto their novel in-group. This argumentation is supported by previous research that has shown that positive self-esteem may even be transferred to a novel in-group that is objectively equivalent to one’s out-group [56], which is also the case in the current experiment. The more overlap there is between the self and the in-group, the stronger the favoritism towards the in-group [57]. Hence, by creating a strong illusion of ownership of a Black virtual body, we facilitate the transfer of positive self-evaluations to the novel in-group; to the extent that the novel in-group is preferred over one’s natural in-group.

Such virtual race transformations may be an effective strategy for combating automatic expressions of racial bias. It goes beyond traditional forms of perspective-taking that requires participants to imagine being a member of the out-group [34]. Instead, it provides the opportunity to actually *experience* an alternative racial identity, and turns people’s natural out-group temporarily into their novel in-group. As demonstrated in the current experiment, this has an immediate impact on participants’ automatic social behaviors in simulated interracial interactions.

It is important to note that what might be thought of as a non-important change as a result of embodiment (i.e., increasing rates of mimicry) may have wide-ranging consequences. Letting participants unconsciously exhibit a reversed racial in-group bias may facilitate the establishment of more positive interracial relationships outside of the VR simulation. While this further ranging implication has yet to be investigated, the current research points into a promising new direction in the still ongoing struggle against racism in many areas of our daily lives.

Finally we remark on some limitations of this study. The ideal experimental design would have included an asynchronous condition, to make doubly sure that the effects were due to the illusion of body ownership. We did not include this because in every study where there has been a visuomotor asynchronous condition the illusion of body ownership was substantially reduced and correspondingly the attitudinal and behavioral consequences were similarly extinguished [5, 6, 39]. Of course we cannot be sure that the same would have occurred with this study, but it is highly likely. Second, another aspect of the ideal experimental design would have been that it would have allowed within group comparisons, i.e., paired scores on individuals embodied in both the Black and White body in counterbalanced order. However, as was found in [58] there can be very strong order effects, and moreover participants are more likely to be able to guess the purposes of the study. Third, although study of the IAT was not the motivation for this work, our finding that there was no change in IAT as a result of embodiment in the Black body was not what we expected prior to the experiment. Therefore the analysis of the impact of liking the virtual partner was post hoc, and should be considered as having generated a hypothesis for a new study rather than as a conclusive result. Finally, the study was carried out only with female participants, a limitation that should be addressed in subsequent work.

## Methods

### Participants and design

The experiment was conducted with 32 White, female students from the University of Barcelona. All but one were aged between 19 and 31 years with one aged 52. The results are not affected by whether or not the individual aged 52 is excluded. We used a 2×2 mixed-factorial design with Own Body as a between-subject factor (Self White and Self Black) and the virtual partner’s race, Other Body, as the within-subject factor (Other White, Other Black). Participants were randomly assigned to one of the racial embodiment conditions. Half were embodied in a White virtual body and the other half in a Black one. All participants interacted with two other virtual characters (one White, one Black), in counter-balanced order. The experiment was approved by the Comissió de Bioètica de la Universitat de Barcelona, and carried out in accordance with the approval. Participants gave written informed consent.

### Virtual environment

The virtual environment was programmed using Unity 3D (version 5.0.4) and displayed using an Oculus Rift (DK2) head-mounted display. It consisted of a simple 3D virtual room equipped with a virtual mirror, which reflected the participant’s virtual body as a mirror image (Fig 1). When participants entered the virtual environment, they were embodied in a life-sized virtual body that visually substituted their real body as seen from their own first person perspective. They would see the virtual body instead of their real body when looking down towards themselves and when looking at their reflection in the virtual mirror. As they moved their real body, they would see their virtual body move synchronously. This was achieved by real-time motion capture from a full-body tracking suit for real-time motion capture, the Xsens MVN system, using techniques described in [59].

During the interaction task, another virtual body was in the room next to the participant and photographs were displayed on the wall in front of both the participant and the virtual character. The participant’s virtual body was positioned so that the other virtual body was always within her visual field–both seen through the mirror or standing to her right.

**Fig 5:**
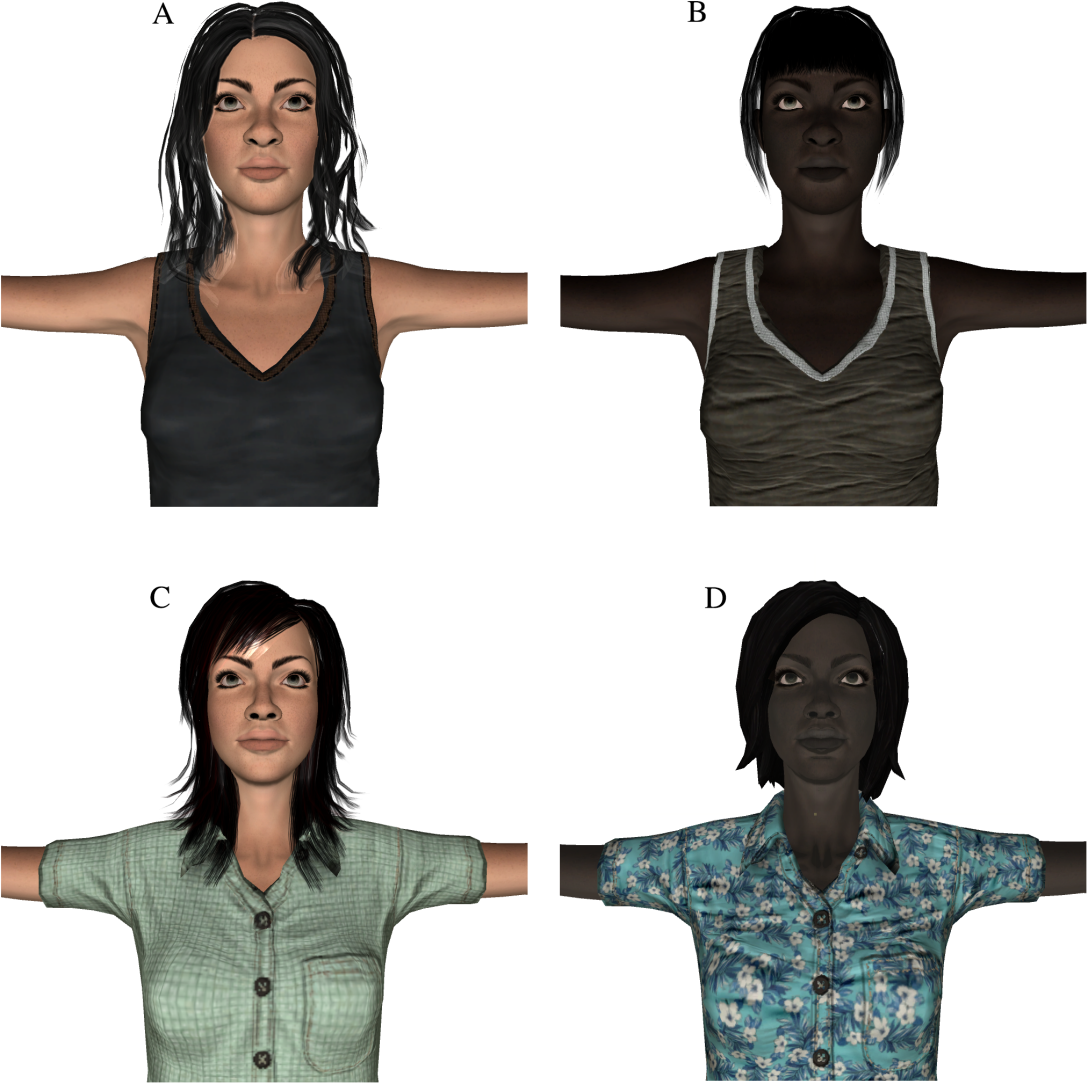
The virtual bodies (A) Own virtual body (White) (B) Own virtual body (Black) (C) Other virtual body (White) (D) Other virtual body (Black).

The virtual bodies were created using Mixamo Fuse (version 1.3). Identical 3D models were used for the two virtual interaction partners (Fig 5). They had the same facial features except for different skin color and hair style, the same body height and width, and wore the same clothes but with variations in color and texture. They were animated in a standardized way using a pre-recorded sequence of body movements from a human actor recorded by the Xsens system. The two versions of participants’ own virtual body (White, Black) were created in the same way based on identical 3D models. However, their body movements were generated in real-time according to participants’ actual body movements.

### Picture description task

The interaction between the participant and their virtual partner consisted of a picture description task; a procedure adapted from previous mimicry experiments [12, 60]. It provides a structured way of interacting with another person without disclosing any personal information, which may unintentionally influence the rate of mimicry. Participants took turns with their virtual partner to describe pictures that appeared on the virtual wall in front of them. We used a set of 12 photographs that display people in different social situations (e.g., shopping, traveling, or playing). The photographs were taken from various websites. The criteria for selection were moderate emotional content and similar amount of portrayed activity, as well as a certain level of ambiguity leaving enough room for interpretation. In each session 6 pictures were displayed (in counter-balanced order across conditions). Each picture was displayed for 1 minute. The task was for the virtual partner and the participant to each express their views about the pictures. They were asked to describe what they could see on the picture (e.g., visual aspects) and/or anything that comes to mind (e.g., what the persons on the photograph might be thinking or feeling). The voice of the virtual partner’s descriptions had been pre-recorded and were played automatically and included automatically generated lip sync derived from the recorded voice.

The sequence of movements were exactly the same for each of the two virtual interaction partners. We used a 3 minute sequence of mimicry triggers (touching hands, arms on hips, scratching arm etc.) about every 5–10 seconds. This 3 minute sequence was repeated once–with smooth transitions from the end of the first to the beginning of the second sequence so that the total interaction time was exactly 6 minutes.

### Response variable details

*Mimicry count*. The extent of mimicry (i.e., how often the participant imitated the other virtual body) was determined by two independent coders based on video recordings of participants’ physical body movements during the picture description task. These videos were synchronized with clips of the other virtual character’s movements, which were played in skeleton view in order to conceal its appearance (see S1 Video). Hence, the coders were blind to the experimental condition. Neither the race of participants’ own virtual body nor the other’s race were visible during coding. Sound was removed from the videos in order not to distract from the nonverbal behaviors. Coded behaviors include: touching face, touching hand, arms on hips, arms down, scratching arm, and shifting weight between left and right foot. Only those cases were considered as mimicry where the participant performed a similar gesture/posture as the virtual partner with a delay of no more than 10 seconds. This is a common practice employed in previous mimicry studies, e.g., [61]. For each mimicry event, one point was added to the mimicry score. When the participant performed one of the target behaviors but the other virtual character did not display this behavior in the previous 10 seconds, it was not scored as a mimicry event. The virtual partner performed a gesture or weight shift every 5–10 seconds, and any similar movement by the participant was marked as mimicry that occurred within the following 10 seconds. For example, the virtual partner may have shifted her weight from the left to the right leg and then folded her hands all within 5 seconds and the participant may have mimicked the hand folding, but not the weight shift. This would have resulted in a count of 1. Had the participant followed both the hand folding and weight shift this would have been counted as 2. Other gestures were very short, such as scratching an arm or the face. Therefore in some cases it would have been possible for 2 different gestures to have been performed by the virtual partner and only one mimicked by the participant. Inter-rater reliability was high in both the first (Cronbach’s alpha = .87) and the second VR session (Cronbach’s alpha = .92). Disagreements between the two coders were resolved by consensus.

*Racial IAT*. Participants’ racial in-group bias was measured using a racial IAT [62] that was used in a previous racial embodiment experiment [6]. The test requires participants to categorize faces of Black and White people as well as positive and negative words. The IAT score is calculated based on the difference in response times and categorization accuracy between bias-congruent trials (White-positive and Black-negative) compared to bias-incongruent trials (Black-positive and White-negative). Higher scores indicate greater racial in-group bias; that is, shorter response times and lower error rates in categorizing congruent trials compared to incongruent trials.

### Procedures

The experiment was conducted in three parts with a 2–3 day interval between sessions. In the first session, participants received information about the experimental procedure and were asked to sign an informed consent form. Then they completed a questionnaire containing general demographic questions as well as questions about their prior experience with VR and video games. Finally, they performed a racial IAT (PreIAT measure). In the second and third sessions, participants were immersed in the VR environment, embodied either in a White or Black virtual body according to their experimental condition. Each of these VR sessions started with a three-minute embodiment phase, which required participants to perform a standardized sequence of movements to become familiar with their virtual body. Pre-recorded instructions were delivered through headphones, to move their head, arms, and legs while looking down towards themselves or looking at their reflection in the virtual mirror. At the end of this phase, textual instructions were presented about the next part of the experiment. Then they engaged in a 6-minutes picture description task, taking turns with their virtual partner describing the pictures that appeared on the virtual wall in front of them. During the task, the virtual partner displayed a pre-recorded sequence of body postures and gestures as potential triggers for imitation, including face and hand touching, arm scratching, arms on hips, arms down, and weight shifts between right and left foot. Participants’ physical body movements were video-recorded during this task in order to count the amount of mimicry later. Immediately after completing the picture description task, participants completed a questionnaire related to body ownership and agency. In addition, they provided explicit evaluations of their virtual partner and their task experience. These additional measures are reported in S1 Text. After the final session the IAT measure was repeated (PostIAT).

### Statistical methods

Stata 14 (www.stata.com) was used for all statistical analysis, and the bar chart with a Stata program [63]. The mixed effects Poisson regression used the Stata function ‘mepoisson’ with fixed effects Own Body and Other Body and random effects over the individuals with the robust standard errors option. The polychoric PCA was carried out with the Stata function ‘polychoricpca’ [64]. The data file is available in S1 Data.

## Supporting information

S1 Text Background data and supplementary analysis. (DOCX)

S1 Video A video showing the major highlights of the experimental study. (MP4)

S1 Data The full data set in Microsoft Excel format. (XLSX)
