# Supplementary material for: Virtual race transformation reverses racial in-group bias
Source: PLoS One. 2017 Apr 24;12(4):e0174965. doi: 10.1371/journal.pone.0174965 (PMC5403166; doi:10.1371/journal.pone.0174965)
Supplement: S1 Text — (DOCX) [file pone.0174965.s001.docx]

Virtual Race Transformation Reverses Racial In-group Bias

Béatrice S. Hasler, Bernhard Spanlang and Mel Slater

Supporting Text S1

**Demographics and prior experience.** Basic demographic information and prior experience with VR and video games were collected in a pre-questionnaire (Table A). The majority of participants were Graduate students (*n* = 27). Four were Master students and one indicated “other.” All except for one participant had Spanish nationality. VR experience was measured using a single item rated on a scale from 1 = no experience to 7 = extensive experience. Video game experience was assessed by hours per week. A further self-assessment of video game playing asked participants to choose the number of times per year that they played. For those in the Self White group 14 out of 16 reported playing at most 20 times per year, compared to 10 out of 16 for those in the Self Black group.

**Table A**. Group Characteristics on Age, previous VR experience and game playing

| **Own Body** | **Age** | **Previous experience with VR, 1=none, 7 = extensive** | **Video games (hours per week)** | **n** |
| --- | --- | --- | --- | --- |
|  | *Mean ± SD* | *Median (IQR)* | *Median (IQR)* |  |
| Self White | 21.9 ± 3.23 | 4 (2.5) | 1(1) | 16 |
| Self Black | 23.8 ± 8.40 | 4.5 (4) | 2(1.5) | 16 |

**Explicit evaluation of the virtual partner.** In both VR sessions, a post-questionnaire was given immediately after completing the picture description task. In addition to the body ownership questions reported in the main analysis, participants were asked to rate their virtual interaction partner with respect a list of questions characterizing their perception of the other virtual character and their relationship (Figure A). Liking, sympathy, intelligence, and similarity were rated on a scale from 1 = not at all to 5 = very much. Connectedness was measured using an adapted version of the Inclusion of the Other in the Self (IOS) scale [1]. A series of pairs of circles was presented with increasing overlap (1 = no overlap; 5 = almost complete overlap). Participants were required to select the image that best represents their relationship to the other virtual character. The results are shown in Figure A.


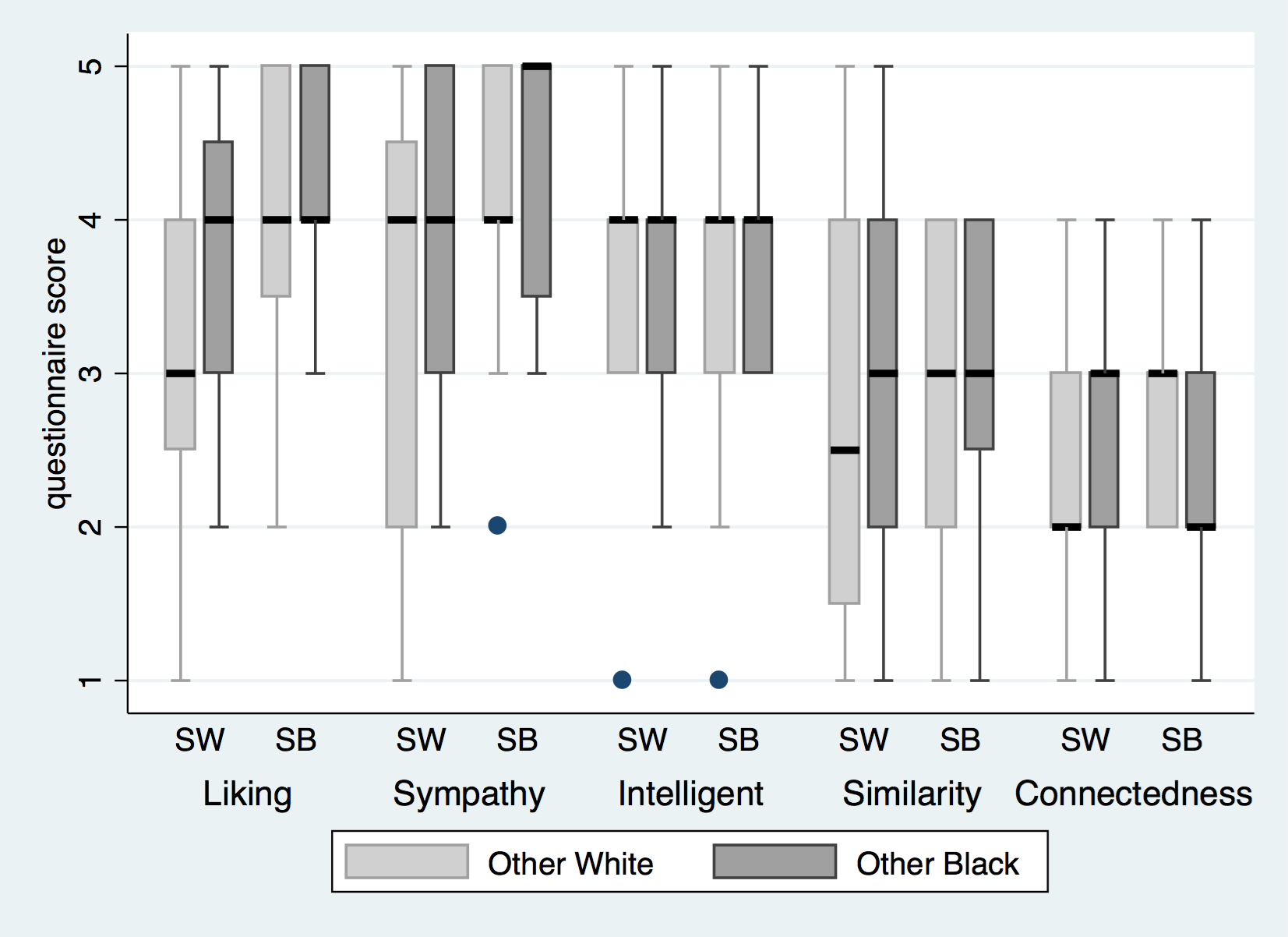


Figure A. Box Plots of Partner Ratings by Own Body
(SW = Self White, SB = Self Black)

In order to test both main effects and possible interaction terms, mixed effects logistic regressions were used for each of these questionnaire responses variables on the model: Own Body + Other Body + Own Body × Other Body. This used the Stata function ‘meologit’, with robust standard errors in order to allow for departures from model assumptions. The interaction term is significant only in the case of Connectedness (P = 0.002). Amongst the other scores the smallest interaction P value was for Liking with P = 0.371. Therefore the interaction term was eliminated for all of the models except for Connectedness. The results are shown in Table B.

Own Body as Black and Other Body as Black results in the highest scores for Liking, with a relatively high median of 4 out of the maximum 5. The only other significant result is for Connectedness, but the medians in all cases are at most 3, which is the neutral point on the scale.

**Table B**. Results of Mixed Effects Logistic Regression.

Each entry shows the z statistic and the corresponding significance level (P).

- Indicates that the corresponding term was not included in the model

The sign of z indicates the direction of relationship between the factor and the response

For each factor White = 0 and Black = 1.

| **Variable** | **Own Body** | **Other Body** | **Own Body × Other Body** |
| --- | --- | --- | --- |
| **Liking*** | 2.11 (0.035) | 1.94 (0.052) | - |
| **Sympathy** | 1.77 (0.076) | 1.23 (0.218) | - |
| **Intelligent** | 0.45 (0.653) | 1.38 (0.169) | - |
| **Similarity** | 0.35 (0.730) | 1.06 (0.288) | - |
| **Connectedness*** | 1.72 (0.085) | 2.42 (0.015) | -3.15 (0.002) |

Liking is particularly important because of the role it played in understanding the results for dIAT. In order to allow for the possibility that the influence of Own Body on Liking, and the influence of Liking on dIAT might affect the apparent influence of Own Body on dIAT we carried out a path analysis. This allows dIAT to be influenced as before by the main effects Own Body and Liking, and the interaction effect of Own Body × Liking. In addition we add a path from Own Body to Liking (Figure B). Now we can examine how this model influences the results. We find that for those with the Other Body as Black the interaction term Own Body × liking is still significant (P = 0.001), and the path from Own Body to liking has P = 0.088. For those with Other Body as White we carry out the same analysis. Again nothing changes in comparison to the results presented in the paper (i.e., none of the paths to dIAT are significant), but as would be expected the path from Own Body to liking has P = 0.019. The conclusion is that adding Liking into this more complex model does not change any of the results reported in the main paper.


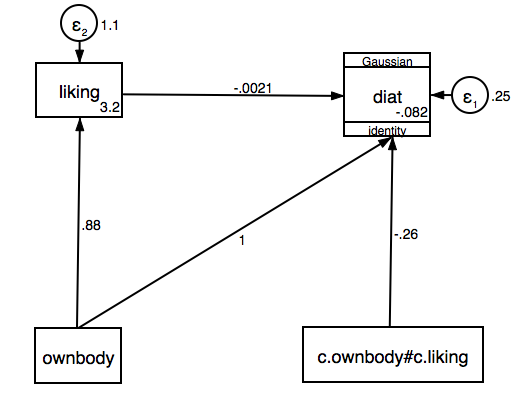


Figure B. The structure of the path diagram

**Subjective task evaluation.** Participants were asked to rate as how difficult they perceived the picture description task and how well they thought they performed in relation to the other character’s performance (Table C). Task difficulty was rated on a scale from 1 = very difficult to 5 = very easy. Task performance was rated on a scale from 1 = ‘I was much better than the other’ to 5 = ‘The other was much better than me,’ with 3 = ‘We were equal.’ The conditions did not differ regarding these ratings.

**Table C**. Medians (IQR) of Subjective Ratings of Task Difficulty and Performance by Condition

|  | **Other White** | | **Other Black** | |
| --- | --- | --- | --- | --- |
| **Own Body** | **Difficulty** | **Performance** | **Difficulty** | **Performance** |
| **Self White** | 4(1) | 3(1) | 4(1.5) | 3.5(1) |
| **Self Black** | 4 (0.5) | 4(1) | 4(2) | 4(1) |

# References

1. Aron A, Aron EN, Smollan D. Inclusion of Other in the Self Scale and the structure of interpersonal closeness. J Pers Soc Psychol. 1992;63(4):596.
